# The place of allogeneic stem cell transplantation in aggressive B-cell non-Hodgkin lymphoma in the era of CAR-T-cell therapy

**DOI:** 10.3389/fmed.2022.1072192

**Published:** 2022-12-06

**Authors:** Luca Castagna, Roberto Bono, Stefania Tringali, Giuseppe Sapienza, Alessandra Santoro, Alessandro Indovina, Vittoria Tarantino, Laura Di Noto, Aurelio Maggio, Caterina Patti

**Affiliations:** ^1^BMT Unit, AOR Villa Sofia-Vincenzo Cervello, Palermo, Italy; ^2^Onco-Hematology and Cell Manipulation Laboratory Unit, Azienda Ospedaliera Riunita (AOR) Villa Sofia-Vincenzo Cervello, Palermo, Italy; ^3^Onco-Hematology Unit, Azienda Ospedaliera Riunita (AOR) Villa Sofia-Vincenzo Cervello, Palermo, Italy; ^4^Transfusional and Transplantation Unit, Azienda Ospedaliera Riunita (AOR) Villa Sofia-Vincenzo Cervello, Palermo, Italy; ^5^Campus of Hematology Franco and Piera Cutino, Azienda Ospedaliera Riunita (AOR) Villa Sofia-Vincenzo Cervello, Palermo, Italy

**Keywords:** allogeneic stem cell transplantation, CAR-T cells therapy, non-Hodgkin lymphoma, refractory, toxicity

## Abstract

Chimeric antigen receptor T (CAR-T) cells are a treatment option for patients with relapse/refractory (R/R) non-Hodgkin lymphoma (NHL), acute lymphoid leukemia and multiple myeloma. To date, diffuse large B-cell lymphoma (DLBCL), mantle cell lymphoma (MCL), follicular lymphoma (FL), and chronic lymphocytic leukemia (CLL) have been successfully treated with CAR-T cells directed against the CD19 antigen. However, when R/R disease persists after several treatment lines, patients with these diseases are often referred to transplantation centres to receive allogeneic stem cell transplantation (ALLO-SCT). ALLO-SCT and CAR-T cells share mechanism of actions, inducing immune effects of *T*-cells (and other cells after transplantation) against lymphoma cells, but they differ in several other characteristics. These differences justify unique positioning of each therapy within treatment algorithms. In this paper, we analyzed the results obtained after ALLO-SCT and CAR-T-cell therapy in patients with aggressive lymphomas (large B-cell lymphoma and MCL) to identify the ideal scenarios in which these 2 immunological therapies should be employed.

## Introduction and background

Although progress has been made in recent years in the treatment and diagnosis of aggressive B-cell lymphomas, particularly diffuse large B-cell lymphoma (DLBCL), with the introduction of monoclonal anti-CD20 antibodies (i.e., rituximab) and consolidation therapy with high-dose chemotherapy in mantle cell lymphoma (MCL), many patients relapse or are considered refractory. The current research era has witnessed an increasing understanding of the molecular abnormalities present in lymphoma cells, and based on this knowledge, more precise therapies have been developed. While these newly identified molecules have mostly been used in treatments for more advanced disease, the efficacy and safety of such treatments suggest that additional agents will be able to be employed in earlier phases of disease.

As such, researchers have long been aiming to develop strategies to manipulate the immune system to fight lymphoma cells, and the first example was allogeneic stem cell transplantation, in which was developed based on studies of leukemia. Several phenomena support the existence of the so-called “graft vs. tumor/lymphoma effect,” such as the lower risk of relapse with ALLO-SCT vs. autologous transplantation, the lower risk of relapse in the presence of graft vs. host disease (GVHD), the higher risk of relapse in cases of mixed chimerism, the increase in efficacy when immunosuppression is withdrawn, and the utility of donor lymphocyte infusion (DLI). However, it is well known that the time of ALLO-SCT can induce severe side effects, leading to a concerning non-relapse mortality (NRM) incidence, which limits its use. From an immunology point of view, donor *T*-cells recognize recipient antigens on lymphoma cells *via* the conventional immunological synapse: HLA-Ag-TCR.

Recently, a new class of immune-active molecules has been developed in clinical trials. Bispecific antibody engagers (BiTEs) enable direct crosstalk of *T*-cells with lymphoma cells independent of the HLA system. Indeed, these antibodies link CD3 molecules on *T*-cells with antigens expressed on lymphoma cells. The first BiTE, used in R/R ALL, was blinatumomab, which is composed of two single-chain variable antibody fragments connected by a flexible linker and is able to link CD3 on *T*-cells with CD19 on leukaemic cells. This linkage enables *T*-cells to kill B cells by granzyme- and FAS/FAS-ligand-mediated mechanisms. Many other BiTEs have been developed, such as glofitamab, mosunetuzumab, odronextamab, epcoritamab, and plamotamab, which are characterized by improved pharmacokinetic proprieties, and additional BiTEs are being developed.

Genetically modified immune cells, both autologous and allogeneic, directed toward specific lymphoma and leukemia antigens are a promising new therapy. The most advanced product is genetically modified autologous T cells, which express a chimeric antigen receptor (CAR) recognizing the CD19 molecule on B cells, both tumoral and normal, and killing them without the need for HLA-mediated antigen presentation. Three CAR-T cells directed against CD19 have been approved in Europe: axicabtagene ciloleucel (axi-cel), tisagenlecleucel (tisa-cel), and lisocabtagene maraleucel (liso-cel).

These CAR-T cells are now used as therapies for R/R DLBCL, primary mediastinal lymphoma, MCL, CLL, and follicular lymphoma. Given its efficacy and safety, CAR-T-cell therapy is replacing ALLO-SCT in clinical practice. However, it should be noted that data from prospective studies are not fully mature.

In this paper, we will briefly present results obtained in several studies of ALLO-SCT and CAR-T cells in lymphoma patients and then present possible scenarios in R/R lymphomas considering the interplay between relevant immunotherapies.

## Post-ALLO-SCT results in LBCL

Clinical results obtained in DLBCL after ALLO-SCT are reported in Tables 1, 2, and from these data, some general conclusions can be made.

**Table 1:** Clinical results after allo-SCT in B-cell NHL.

**Authors**	***N***	N*	**Median Age y**	**Median** **CT lines**	**Previous HDC**	**Disease status**	**Donor**	**CTX**	**OS**	**PFS**	**Relapse**	**NRM**
Robinson et al. (1)	188	62	46	3 (1–5)	29%	CTS 73%	MRD 91% MUD 9%	RIC 90%	46%@2 y	12%@2 y	47%@2 y	36%@2 y
Corradini et al. (2)	170	61	51	3 (1–6)	49%	CTS 77%	MRD 100%	RIC 100%	69%@3 y	46%@3 y	31%@3 y	15%@3 y
Rezvani et al. (3)	68	16	54	6 (1–19)	44%	CTS 63%	MRD 55% MUD 45%	RIC 100%	45%@3 y	35%@3 y	41%@3 y	25%@3 y
Thomson et al. (4)	48	48	46	5 (2–7)	71%	CTS 83%	MRD 81% MUD 19%	RIC 100%	47%@4 y	48%@4 y	33%@4 y	32%@4 y
Hamadani et al. (5)	46	18	46	3 (3–8)	0	CTS 0%	MRD 88% MUD 15%	RIC 93%	38%@5 y	38%@5 y	50% PD 25% SD	43%@100 d PD 9%@100 d SD
Sirvent et al. (6)	68	68	48	2 (1–5)	79%	CTS 83%	MRD 82% MUD 18%	RIC 100%	49%@2 y	44%@2 y	41%@2 y	23%@2 y
Van Kampen et al. (7)	101	101	46	3 (2–6)	100%	CTS 74%	MRD 72% MUD 28%	RIC 64% MAC 36%	52%@3 y	43%@3 y	30%@3 y	28%@3 y
Rigacci et al. (8)	165	165	46	/	/	CTS 55%	MRD 65% MUD 35%	RIC 70%	39%@5 y	31%@5 y	67%@5 y	28%@1 y
Bacher et al. (9)	396	396	48–54	/	18–51%	RIC CTS 35% MAC CTS 40% NMA CTS 35%	/	RIC 36% MAC 41% NMA 22%	RIC 27%@3 y MAC 21% NMA 29%	RIC 23%@3 y MAC 19% NMA 27%	RIC 26%@3 y MAC 26% NMA 28%	RIC 42%@3 y MAC 55% NMA 34%
Hamadani et al. (10)^£^	533	533	46–53	3–4	15–38%	CTS 0%	MRD 48% MUD 24% mMUD 11%	RIC 42%	28%@3 y	23%@3 y	35%@3 y	42%@1 y
Bouabdallah et al. (11)^§*II*^	31	14	57	3 (2–4)	96%	CTS 100%	MRD 66% MUD 34%	RIC 100%	80%@2 y	80%@2 y	7%@2 y	13%@2 y
Glass et al. (12)^§∧^	84	61	48	4 (3–6)	52–55%	CTS 45%	MRD 27% MUD 40% mMUD 31%	MAC 100%	52%@1 y	45%@1 y	29%@1 y	10%@1 y
Fenske et al. (13)	503	503	52	4 (1–7)	100%	CTS 74%	MRD 50% MUD 23% mMUD 26%	RIC 75% MAC 25%	37%@3 y	31%@3 y	38%@3 y	30%@1 y
Dodero et al. (14)^§^	121	35	52	/	61%	CTS 97%	MRD 55% MUD 28% mMUD 17%	RIC 100%	52%@3 y	40%@3 y	27%@3 y	21%@3 y
Kawashima et al. (15)	60	60	55	4 (2–9)	32%	CTS 64%	MRD 25% MUD 22% mD 28% CB 20%	RIC 93%	42%@2 y	59%@2 y	60% DEL 20% noDEL	22% DEL 9% noDEL
Dreger et al. (16)°	1,438	1,438	55–58	/	42–62%	CTS 75–82%	MRD 36% MUD 53% HAPLO 10%	RIC 100%	MRD 50%@3 y MUD 43–46%@3 y HAPLO 46%@3 y	MRD 37%@3 y MUD 36%@3 y HAPLO 38%@3 y	MRD 47%@3 y MUD 38–34%@3 y HAPLO 41%@3 y	MRD 17%@3 y MUD 26–30%@3 y HAPLO 22%@3 y

HDC, high-dose chemotherapy; CT, chemotherapy; N^*^, N of patients with DLBCL; CTX, conditioning regimen; CTS, chemosensitive; mD, mismatched donor; MRD, matched related donor; MUD, matched unrelated donor.

In Hamadani et al. (5), patients were analyzed based on disease status at ALLO-SCT: progressive disease (PD) vs. stable disease (SD).

In Hamadani et al. (10), patients with DLBCL and grade III follicular lymphoma had a better outcome. In this study, a myeloablative conditioning regimen was used in a high percentage of patients. Though this reduced the relapse rate, the PS was worse than that in the RIC group because of the higher NRM incidence (53% in the MAC cohort).

In Kawashima et al., the results were analyzed based on the double expressor subtype.

In Thomsom et al. (4) all patients received in vivo T-cell depletion using CAMPATH.

In Rezvani et al. (3) patients with transformed NHL were included.

In Bacher et al. (9) the authors analyzed the impact of the intensity of CTX on clinical outcomes. The NRM incidence was higher after MAC, while the relapse rate was higher with less intensive CTX regimens, explaining the similar PFS and OS.

In van Kampen et al. (7) the NRM incidence was higher using MAC.

^§^Prospective trials.

^II^In this trial, CTX included Zevalin.

^∧^In this trial, the NRM incidence was reported for patients receiving MRD or MUD transplants with ATG.

°In this study, the MUD group was separated based on whether GVHD prophylaxis was applied with or without in vivo T-cell depletion.

^£^In this study, 226 patients had DLBCL, and 207 had G3 follicular lymphoma.

**Table 2:** Acute and chronic GVHD rates.

**Authors**	***N***	N*	**Grade 2–4 aGVHD**	**cGVHD**
Robinson et al. (1)	188	62	25%	NR
Corradini et al. (2)	170	61	35%	49%
Rezvani et al. (3)	68	16	63%	47%
Thomson et al. (4)	48	48	17%	22%
Hamadani et al. (5)	46	18	43%	75%
Sirvent et al. (6)	68	68	39%	41%
Van Kampen et al. (7)	101	101	33%	42%
Rigacci et al. (8)	165	165	27%	NA
Bacher et al. (9)	396	396	RIC 43% MAC 43% NMA 44%	RIC 37% MAC 41% NMA 37%
Hamadani et al. (10)	226	226	30%	35%
Bouabdallah et al. (11)^§^	31	16	27%	14%
Glass et al. (12)^§^	84	61	42%	41%
Fenske et al. (13)	503	503	36%	47%
Dodero et al. (14)^§^	121	35	22%	44%
Kawashima et al. (15)	60	60	64%	40%
Dreger et al. (16)	1,438	1,438	MRD 32% MUD 32–42% HAPLO 34%	MRD 48% MUD 27–57% HAPLO 18%

NR, not reported.

^§^Prospective trials.

The survival results vary substantially, ranging from 12 to 80% for progression-free survival (PFS) and 28 to 80% for overall survival (OS). Toxicity is also variable, as the NRM incidence ranges from 9 to 55%, the acute GVHD incidence ranges from 17 to 64%, and the chronic GVHD (overall) incidence ranges from 14 to 75%. This heterogeneity has several explanations. For example, some studies (1–3, 5, 10–12, 14) included subtypes other than DLBCL. In addition, most of the studies were retrospective or registry-based, which can lead to selection bias, and there were differences in transplant characteristics between the studies, such as donor characteristics and the intensity of conditioning regimens.

It is well known that immunotherapy is more active in indolent/follicular lymphoma (17) than in more aggressive subtypes such as DLBCL, MCL and T-cell lymphomas. Indeed, this was evident in the EBMT study (1) and in an Italian study (2), in which patients with indolent lymphoma achieved longer survival.

Some studies included patients with chemorefractory disease at the time of ALLO-SCT. This is important because the disease status before ALLO-SCT is consistently reported to be one of the most reproducible prognostic factors for survival (Table 3). Three studies are interesting in this regard because they analyzed only patients with chemorefractory disease (5, 10, 12). Two were retrospective analyses. The first was from a single centre and included 46 lymphoma patients with chemorefractory disease who received ALLO-SCT from 1988 to 2007. Only 16 DLBCL cases were included. As reported in Table 1, PFS and OS were better in patients with stable disease than in patients with progressive disease at the time of ALLO-SCT. The latter group of patients were more likely to relapse and/or die, with a substantial difference in survival (5). The second study, which included 226 DLBCL (and 207 grade III follicular lymphoma) cases, was a registry-based analysis. Most of the donors received a transplant from a MUD and had MRD, but 11% of patients received transplantation from a mismatched unrelated donor (mMUD), and 58% of patients received a myeloablative conditioning regimen. The outcomes are reported in Table 1. It is interesting to note that in a multivariate analysis, the NRM incidence was lower for grade III FL; in addition, with RIC, the PFS and OS were higher and the relapse rate was lower in grade III FL. More intensive conditioning regimens were associated with a reduced risk of relapse (10). In a phase 2 randomized study, Glass et al. included patients with aggressive lymphoma (DLBCL, 61 out of 84 patients) who relapsed after high-dose chemotherapy (53%) and were refractory to the first CT line (57%) or who relapsed within <12 months (16%). All patients received a MAC regimen. Only 45% showed chemosensitive disease at the time of ALLO-SCT. In this unfavorable group of patients, the 1-y PFS and OS were 52 and 45%, respectively, with a better outcome when the patient had achieved MRD negativity or a MUD and a conditioning regimen containing ATG were used (12). Notably, the 1-y NRM incidence was only 10%. Interestingly, the inclusion criteria of this prospective study are the same as those used in some phase 2 studies of patients treated with CAR-T cell (18, 19) and thus allow an indirect comparison of CAR-T-cell therapy and ALLO-SCT. The CIBMTR reported the outcome of DLBCL patients relapsing after HDC, and overall, the 3-y PFS and OS were 31 and 37%, respectively. In that paper, the authors identified some prognostic factors (time to relapse after HDC, disease status at the time of ALLO-SCT, and PS) and developed a scoring model to predict survival (13). Of note, the model can predict the outcome of patients treated with CAR-T cells (20).

**Table 3:** Risk factors (RF) for the major outcomes identified in selected studies.

**Authors**	***N***	N*	**Disease status**	**Donor**	**RF OS**	**RF** **PFS**	**RF Relapse**	**RF** **NRM**
Robinson et al. (1)	188	62	CTS 73%	MRD 91% MUD 9%	Chemosensitive	Chemosensitive	Chemosensitive	Age > 50 y
Corradini et al. (2)	170	61	CTS 77%	MRD 100%	Chemosensitive Disease histotype Previous HDC Severe aGVHD	Chemosensitive Disease histotype Severe aGVHD	Chemosensitive Disease histotype	
Van Kampen et al. (7)	101	101	CTS 74%	MRD 72% MUD 28%	TtR after HDC > 12 M High LDH	TtR after HDC > 12 M High LDH BM	Disease status	Age > 45 y BM TtR after HDC > 12 M
Rigacci et al. (8)	165	165	CTS 55%	MRD 65% MUD 35%	CR > PR > other	CR > PR > other MRD		
Bacher et al. (9)	396	396	RIC CTS 35% MAC CTS 40% NMA CTS 35%	/	KPS Disease status Year of ALLO-SCT	KPS Disease status Year of ALLO-SCT	Disease status RIC/NMA	KPS Disease status Year of ALLO-SCT Donor type MAC better
Hamadani et al. (5)	533	226	CTS 0%	MRD 48% MUD 24% mMUD 11%	DLBCL	DLBCL	RIC (vs. MAC) DLBCL Previous HDC	DLBCL Donor type
Glass et al. (12)	84	61	CTS 45%	MRD 27% MUD 40% mMUD 31%	/	aGVHD 0–1 mMUD Refractory No ATG	/	/
Fenske et al. (13)	503	503	CTS 74%	MRD 50% MUD 23% mMUD 26%	PS <80% Chemoresistant TtR After HDC <12 M MAC	PS <80% Chemoresistant TtR After HDC <12 M MAC	PS <80% Chemoresistant TtR After HDC <12 M	Chemoresistant Donor type
Dreger et al. (16)	1,438	1,438	CTS 75–82%	MRD 36% MUD 53% HAPLO 10%	CR > PR > SD/PD Age HCT-CI	CR > PR > SD/PD	MUD better	MRD better Age HCT-CI

TtR, time to relapse.

The conditioning regimens were mostly reduced intensity or non-myeloablative in nature, but in some studies (7, 9, 13, 15), some patients were treated with intensive conditioning regimens, suggesting that the intensity of the conditioning regimen might be a relevant factor. For example, should a MAC regimen be used in patients with advanced disease to encourage the graft vs. lymphoma effect? In the prospective German study DSHNHL R3, all patients received a MAC regimen consisting of fludarabine, busulfan (3–4 days) and cyclophosphamide. More than half of the patients had refractory disease at the time of ALLO-SCT, and the median age was 48 years. Though the age limit for inclusion was 65 years, the oldest included patient was 57 years. The 1-y NRM incidence was 10%, and interestingly, the 1-y PFS was 45%, and the 1-y OS was 52% (12). In a retrospective study from CIBMTR comparing MAC, RIC, and non-myeloablative conditioning (NMAC) regimens in DLBCL, and as reported in other studies (7, 13, 15), the reduced risk of relapse/progression with MAC was offset by a higher risk of early and late NRM, and consequently, the survival was not different (9). In a recent analysis, including 1,823 NHL patients (every histology), Ghosh et al. compared the outcomes based on the intensity of the RIC regimen. Again, the most intensive regimen, namely, fludarabine plus melphalan 140 mg/m2, showed a less favorable profile in terms of NRM, without any improvement in relapse risk (21). This study suggests that more intensive conditioning regimens do not lead to superior results in lymphoma patients. However, we think that younger patients (maybe <45 years), patients who have not been treated with HDC or autologous stem cell therapy, patients without comorbidities, and patients who are not in complete remission could benefit from a MAC regimen.

Another variable that could affect the outcomes is the donor type. Although an HLA-identical sibling (HLAid sib) or matched unrelated donor (MUD) was usually used in the past, with the advent of the PTCY platform, haploidentical donors can be used. This advance has changed the treatment landscape, allowing more patients to be transplanted. The impact of donor type (HLAid sib vs. MUD) was analyzed in most of the studies reported in Table 1. In some of the studies, donor type did not impact survival or NRM incidence (3, 4, 6, 7, 12, 14), while in others, an MUD transplant was associated with a higher incidence of NRM (8–10, 13), lower PFS (8) or higher GVHD incidence (1). Recently, a joint retrospective analysis from CIBMTR and EBMT of patients with DLBCL receiving transplantation from an HLAid sib, an MUD with or without T-cell depletion (TCD), or a haploidentical donor. This study confirmed that the survival rate after transplantation from a haploidentical donor and administration of cyclophosphamide posttransplantation was similar to that observed after transplantation from an HLAid sib or MUD. It was confirmed that the graft-relapse free survival (GRFS) was better with haploidentical donors due to the low incidence of chronic GVHD. Furthermore, in this study, the NRM incidence of patients receiving an MUD transplantation without TCD was significantly higher (16).

A relevant clinical aspect of ALLO-SCT is the age of patients with NHL (DLBCL 30%); ALLO-SCT is a valid therapeutic option in elderly patients. In a CIBMTR study, Shah et al. showed that the 4-year relapse rate, PFS and OS were similar in a cohort of older patients (median age 68 years) compared to a cohort of younger patients (median age 60 years). Only the 4-year NRM was slightly higher in the older patient cohort (22).

Furthermore, immunological activity against lymphoma cells can be reflected in the relapse rate. Indeed, B and *T*-cells in lymphoma tissue express antigens recognized by donor T cells. Table 1 shows the relapse rate observed in several clinical studies. Because most of the studies were retrospective and thus may have selection bias that may have influenced the results, the relapse rate ranges from 7 to 60%. It is not surprising that the disease status at the time of ALLO-SCT is the main factor related to this phenomenon. This pattern was also observed in the study by Hamadani et al. (5), in which only patients with SD or PD were included, and in the study by Bouabdallah et al. which included only CR patients (11). In these 2 studies, the relapse rates were 25% when in SD, 50% in PD, and 7% in CR, respectively. Another factor could be the intensity of conditioning regimen. As described above, high-intensity conditioning regimens can have more activity against lymphoma, but their toxic side effects can be prohibitive. In conclusion, the disease status before ALLO-SCT is the most important factor related to relapse.

Finally, after allo-SCT, only one study reports that DE DLBCL receiving allo-SCT showed inferior PFS linked to higher relapse incidence compared to no-DE lymphomas (15).

## Results after CAR-T-Cell therapy in LBCL

CAR-T-cell therapy has changed the treatment landscape for many patients with relapsed or refractory aggressive B-cell lymphomas. Since 2017, when the three commercial CAR-T-cell products were approved, many clinical trials and data have been reported.

The pivotal phase 2 prospective studies ZUMA-1 (18), JULIET (19) and TRANSCEND (23) for axicabtagene ciloleucel (axi-cel), tisagenlecleucel (tisa-cel) and lisocabtagene maraleucel (liso-cel), respectively, enrolled heavily pretreated patients who relapsed after or were refractory to at least two prior lines of standard therapy and autologous stem cell transplantation. In these studies, the histology-related inclusion criteria were different; in general, the studies included high-grade B-cell lymphoma (HGBCL) with or without translocations of MYC and BCL2 and/or BCL6 (double/triple-hit lymphoma) and transformed follicular lymphoma (tFL). Both ZUMA-1 and TRANSCEND also included patients with R/R primary mediastinal B-cell lymphoma (PMBCL). Only TRASCEND included patients with transformed diffuse large B-cell lymphoma arising from indolent histologies other than FL or FL3B (2, 3) and those with secondary CNS involvement or who had received prior ALLO-SCT. Table 4 shows the main results from the prospective and retrospective studies. The follow-up was shortest in the TRANSCEND trial, with data from a median follow-up of 18.8 months (vs. 27.1 months in ZUMA-1 and 32.6 months in JULIET). Even though there were differences in the inclusion criteria across the 3 studies, the ORR (ranging from 52 to 74%) and CR rate (ranging from 40 to 54%) were comparable across age and tumor histology subgroups. Interestingly, almost 40% of the refractory patients in these studies were disease free 3–4 years after infusion.

**Table 4:** Main clinical results from prospective and retrospective studies of aggressive lymphomas.

**Authors**	***N***	**Median age**	**Phase**	**Drug**	***N* CT lines**	**ORR**	**FU (months)**	**PFS** **(months)**	**OS (median)**
Neelapu et al. (18)	101	58	P 1–2	Axi-cel	3	74% (CR 54%)	27.1	5.9	NR
Schuster et al. (19)	115	56	P 2	Tisa-cel	3	52% (CR 40%)	32.6	NR	NR
Jacobson et al. (24)	122	62	R	Axi-cel	2–3	70% (CR 50%)	10.4	4.5	NR
Abramson et al. (23)	269	63	P 1	Liso-cel	3	73% (CR 53%)	12–17.5	6.8	NR
Nastoupil et al. (25)	298	60	R	Axi-cel	3	82% (CR 63%)	12.9	8.3	NR
Pasquini et al. (26)	410	65	R	Tisa-cel	4	62% (CR 49%)	11.9		
Lamure et al. (27)	60	64	R	Axi-cel Tisa-cel	2–3	63% (CR 40%)	6.9	3.1	12.3
Iacoboni et al. (28)	72	60	R	Tisa-cel	3	BOR 60% (CR 32%)	14.1	3	10.7
Sehgal et al. (29)	61	74	P 2	Liso-cel	n.a.	80% (CR 54%)	12.3	9.03	
Bastos-Oreiro et al. (30)	204	axi-cel 54 tisa-cel 56	R	Axi-cel Tisa-cel	2	60%	10 in axi-cel; 14 in tisa-cel	8.5 axi-cel; 4.6 tisa-cel	NR axi-cel; 11.7 tisa-cel
Betghe et al. (31)			R	Axi-cel Tisa-cel					
Kwon et al. (32)	307		R	Axi-cel Tisa-cel	2	57% (38%)	9.2	4.8	11.7
Bachy et al. (33)	518	63	R	Axi-cel Tisa-cel	2				

MA, metanalysis; NR, not reached; NA, not applicable; ORR, overall response rate; P, prospective; R, retrospective.

Clinical trials have stringent eligibility criteria, and the outcomes observed in these trials may not be observed in real-life clinical practice. Several retrospective studies of the use of commercial CAR-T-cell products have been published (Table 4). Some general conclusions can be made from real life studies. Overall, in the real world, the groups of patients treated with CAR-T cells are less refined than those in prospective studies, but the clinical results are similar in terms of ORR (ranging from 59 to 84%), CR rate (ranging from 32 to 65%) and survival. In the real life studies compared with the clinical trials, the vast majority of patients had DLBCL, the median age was higher, there was a greater number of patients with ECOG PS > 2, and just over half of the patients treated with axi-cel received bridging therapy (BT), which was not allowed in ZUMA-1. The role of bridging therapy is unclear. CAR-T-cell therapy is typically used for patients who are resistant to chemotherapy, and thus, BT should improve disease control prior to CAR-T-cell infusion. On the other hand, it is possible, that patients treated with BT may have inferior outcomes because they have more aggressive and rapidly progressive disease or because the BT itself confers additional treatment toxicity or immunosuppression (34). Conventional BT have not been as effective as expected, and in a recent retrospective trial analyzing the impact of different BT before axi-cel infusion, it was clear that patients who received systemic BT showed more advanced and aggressive disease, and their survival was lower than that of patients who did not receive BT. Furthermore, the studies show that patients who received radiotherapy had longer PFS than patients who received systemic therapy as BT (35). Recently, the Spanish groups GELTAMO and GETH published a comparison of real-world CAR-T-cell therapy with standard of care (SOC) treatment for refractory large B-cell lymphoma, and their results confirmed the higher efficacy of CAR-T-cell therapy than SOC, showing longer PFS and OS in the CAR-T-cell therapy group independent of other prognostic factors. In the CAR-T cell therapy cohort, CAR-T-cell therapy type (axi-cel better), unfavorable R-IPI at LD, no previous ASCT, and higher Haematopoietic Cell Transplantation-specific Comorbidity Index (HCT-CI) before lymphodepletion (LD) adversely influenced PFS while CAR-T-cell therapy type (axi-cel better), unfavorable R-IPI at LD, ECOG-PS 2–4 before apheresis, primary refractory disease, and higher HCT-CI before LD impacted OS in the multivariate analysis (30). The efficacy of tisa-cel and axi-cel was assessed in 3 recent retrospective studies. In the first single-centre study, axi-cel was clearly more toxic than tisa-cel or a homemade 41-BB CAR-T-cell product, but the ORR was influenced by absolute lymphocyte count (ALC) before leukapheresis, with axi-cel being more active when ALC was high (36). In a second study, 307 patients were analyzed (152 who received axi-cel vs. 155 who received tisa-cel) in a multicentre setting. The patient characteristics were well balanced, and while the ORR, duration of response (DOR), PFS and OS were not significantly different between the treatments, the incidence of ICANS was higher while the CRS rate was similar after axi-cel (32). Different findings were reported in the second study (33), in which a large number of patients included in the DESCART national registry were analyzed (209 who received tisa-cell vs. 209 who received axi-cel) using propensity score matching to reduce differences in variables associated with outcomes. The overall CRS incidence was higher after axi-cel than after tisa-cell (86.1 vs. 75.6%), but the severe CRS incidence was similar (9.1 vs. 5.3%); in addition, the overall and severe ICANS incidences were higher after axi-cel than after tisa-cel (48.8 vs. 22 and 13.9 vs. 2.9%, respectively), as was the rate of cytopenia. The ORR and CR rate were significantly higher for the axi-cel group than for the tisa-cel group (80.4 vs. 66 and 60.3 vs. 42.1%, respectively), and the 1-year PFS and OS were longer for the axi-cel group than for the tisa-cel group (46.6 vs. 33.2 and 63.5 vs. 48.8%, respectively). Survival was confirmed to be better in the axi-cel subgroups with either age ≥70 years or bulky disease (33). These data confirmed the initial results from the matching adjusted indirect comparison (MAIC) trial (37).

Meng et al. published a meta-analysis of the safety and efficacy of CAR-T cells. Overall, the authors did not find remarkable differences in the terms of ORR, CR rate, or survival, as reported in Table 5 (38).

**Table 5:** Clinical results from the metanalysis (38).

		**Axi-cel**	**Tisa-cel**	**Liso-cel**
All patients	Severe CRS	13%	9%	2%
	Severe ICANS	31%	8%	10%
DLBCL	ORR	70%	75%	72%
	CR rate	52%	40%	52%
Primary mediastinal	ORR	62%	/	/
	CR rate	58%	/	/
High grade BCL	ORR	88%	/	76%
	CR rate	/	/	61%

BCL, B-cell lymphoma; DLBCL, diffuse large B-cell lymphoma; ORR, overall response rate.

CAR-T-cell infusion is associated with specific side effects that result from on-target off-tumor activity. The most frequent toxicities are cytokine release syndrome (CRS), immune effector cell-associated neurotoxicity syndrome (ICANS), and prolonged cytopenias, and less frequently, B-cell aplasia and hypogammaglobulinemia, infections, tumor lysis syndrome and infusion-related immune reactions are described. Table 6 shows the toxicities after CAR-T-cell treatment. It is difficult to compare toxicities across the studies because different scales were used, and there were more toxicities in the first years after commercial CAR-T cell product administration. Notably, newer and more refined grading criteria for CAR-T-cell therapy-associated neurological events (NEs) are now available, such as the ASCT consensus criteria for ICANS, which is currently widely used for reporting NEs in real-world studies (39, 40). In the prospective studies, the cumulative incidences of CRS and ICANS seem to be higher than those in real-life studies, probably because of the early use of tocilizumab and steroids and greater confidence of the clinicians. In general, axi-cel was associated with a significantly higher risk of CRS and severe neurotoxicity due to the rapid and massive T-cell expansion linked to the costimulatory moiety CD28, which is not seen with the 41-BB costimulatory moiety used for tis-cel and liso-cel. Furthermore, the frequency and severity of CRS and NEs were higher in patients with high tumor volume, and patients were more likely to experience a severe (grade > 3) NE after receiving > 5 prior lines of therapy (41–43). In a real-life German study, the NRM incidence at 2 years was significantly higher after axi-cel than after tisa-cel therapy (10.4 vs. 3.5%, respectively) (31).

**Table 6:** CRS and ICANS incidences in clinical studies.

	**CRS**						**ICANS**						**Cytopenia**		
	**Axi-cel**		**Tisa-cel**		**Liso-cel**		**Axi-cel**		**Tisa-cel**		**Liso-cel**		**Axi-cel**	**Tisa-cel**	**Liso-cel**
	**Any**	**Severe**	**Any**	**Severe**	**Any**	**Severe**	**Any**	**Severe**	**Any**	**Severe**	**Any**	**Severe**			
Neelapu et al. (18)	93%	13%					64%	28%					84%		
Schuster et al. (19)			58%	22%			70%	35%	21%	12%				44%	
Jacobson et al. (24)	93%	16%											/		
Abramson et al. (23)					42%	2%					30%	10%			63%
Nastoupil et al. (25)	91%	7%					68%	37%					/		
Pasquini et al. (26)			45%	4.5%					18%	5%				18%	
Iacoboni et al. (27)			71% 5%						15%	1%				/	
Bastos-Oreiro et al. (28)	90%	8%	65%	4%			42%	16%	16%	5%			/	/	
Bethge et al. (31)	81%	10%	65%	13%			76%	28%	40%	12%			17%		
Kwon et al. (32)	82%	8%	73%	6%			42%	16%	18%	5%			10%	15%	
Bachy et al. (33)	86%	5.3%	75%	9.1%			48.8%	13.9%	22%	2.9%			64.6%	39.2%	

Severe toxicities were defined as those ≥ grade 3.

Table 7 shows the factors predictive of response and survival in different studies, both retrospective and phase 1–2. There are many differences, and there is no overlap of predictive factors, but in general, age; factors related to the disease, such as high disease burden and primary refractory disease; and higher IPI were associated with a worse outcome. Some studies found a correlation between CAR-T-cell expansion *in vivo* with the duration of response, with the strongest correlation demonstrated for acute lymphoblastic leukemia. Overall, the biological characteristics of lymphoma cells did not impact the response, i.e., DH/TH subgroups showed the same sensitivity to CAR-T-cell therapy. However, the ORR (34%) and survival (1-y survival rate 44%) in p53-mutated lymphomas were significantly lower than those in unmutated lymphoma (46). In that study, primary refractory disease and SD or PD at infusion were also predictive factors of lower ORR.

**Table 7:** Pre-CART and post-CART factors predicting response and survival after CAR-T-cell therapy identified in clinical trials in aggressive lymphomas.

**Authors**	**Overall response rate**	
	**Pre-CART**	**Post-CAR-*T*-cell therapy**
Neelapu et al. (18)	None	CAR-T-cell expansion
Schuster et al. (19)	None	None
Schuster et al. (44)	LDH levels, G3–4 thrombocytopenia	None
Jacobson et al. (24)	High tumor burden, ferritin, LDH levels	-higher peak CAR-T-cell:TB ratio -CAR-T-cell expansion -Better if high CCR7–CD45RA–T cells
Abramson et al. (23)	None	None
Nastoupil et al. (25)	Age > 60 y, LDH levels before LD	None
Pasquini et al. (26)	None	None
Vercellino et al. (45)	None	None
Locke et al. (41)	High tumor burden, IL6 levels, CRP, Day 0 IL-15, interferon-g in coculture	CAR-T-cell expansion
Lamure et al. (27)	Advanced age, refractoriness to previous treatment, multiple previous lines of treatment	None
Iacoboni et al. (28)	Sex (females did better), higher IPI	None
Shouval et al. (46)	P53 mutation, refractoriness and SD/PD at infusion	None
Bastos-Oreiro et al. (30)	None	
Betghe et al. (31)	IPI > 2, high LDH levels	
	**Survival**	
	**Pre-CART**	**Post-CART**
Neelapu et al. (18)	None	None
Schuster et al. (19)	None	None
Schuster et al. (44)	None	None
Jacobson et al. (24)	Day 0 CRP > 30 mg/L	None
Abramson et al. (23)	None	None
Nastoupil et al. (25)	PFS: Bilirubin levels, LDH levels, ECOG, sex OS: Bilirubin levels, LDH levels, ECOG, sex, disease status, BT	None
Pasquini et al. (26)	None	None
Vercellino et al. (45)	CRP, END > 2, MTV > 41%	None
Locke et al. (41)	High tumor burden, LDH levels, IL6, effector: target ratio, number of infused CD8 T cells, CCR7-CD45RA-T cells	-higher peak CAR T cell:TB ratio
Lamure et al. (27)	Female gender, aaIPI at the time of infusion	None
Iacoboni et al. (28)	Primary refractory disease [HR: 2.24 (95%CI1.20–4.18), *p* = 0.01] and high LDH	None
Shouval et al. (46)	P53 mutation, refractoriness and SD/PD at infusion, LDH levels	None
Bastos-Oreiro et al. (30)	Previous HDCT, primary refractoriness, ECOG PS pre-apheresis, type of CAR-T cell, BT, HCT-CI, R-IPI	None
Betghe et al. (31)	Nonresponse to bridging therapy, elevated LDH, poor PS	None
Kwon et al. (32)	PFS: LDH levels before apheresis, ECOG PS ≥2 before LD therapy OS: high LDH at apheresis, ECOG PS ≥2 at apheresis, progressive disease at apheresis, ECOG PS ≥2 before LD	None

ORR, overall response rate; R, retrospective; P, prospective; LD, lymphodepletion; END, extranodal disease; CRP, C-reactive protein; HDCT, high-dose chemotherapy; BT, bridging therapy, R-IPI, Revised International Prognostic Index; PS, performance status.

In conclusion, CAR-T cells are extraordinarily active against advanced, aggressive lymphomas, even if disease control cannot be achieved before infusion.

## Results after ALLO-SCT in MCL

Mantle cell lymphoma is still considered an incurable disease, even though the survival of patients has improved in recent years. In facts, the survival of transplant-eligible patients with advanced MCL is almost 8 years (47). Treating R/R MCL is a clinical challenge, and Bruton kinase (BTK) inhibitors or anti-Bcl2 agents (venetoclax) can achieve a high objective response rate, though the survival is short and unsatisfactory. Clinical (progression of disease within <24 months after first-line treatment), histological (high Ki67, blastoid morphology), and molecular (p53 mutation) factors can identify subgroups of patients likely to have unfavorable outcomes (48).

R/R MCL can be treated with a BTK inhibitor +/− venetoclax or other conventional combinations (i.e., bendamustine + cytarabine + cyclophosphamide, BAC), and responding patients can be considered for immunotherapy. Before the advent of CAR-T-cell therapy, ALLO-SCT was frequently used as immunotherapy. Table 8 summarizes the clinical results obtained in the last 20 years from studies using ALLO-SCT in RR MCL. The majority of patients relapsed or progressed after receiving AUTO-SCT. In most of the studies reported in Table 8, most patients had experienced a CR or PR just before ALLO-SCT (range 54 to 100%). As expected, the disease status at the time of ALLO-SCT is an important prognostic factor for survival (49–52), and survival is significantly better in patients with a favorable disease status. However, the OS rate varies widely, ranging from 13 to 73%, indicating a strong selection bias. Of particular interest is the CIBMTR study, which included only patients with chemorefractory disease at ALLO-SCT (53), because these patients are similar to the patients that were included in the CAR-T-cell trials. RIC or MAC were used as conditioning regimens, and HLAid siblings or unrelated donors were used. The 3-y OS, PFS, relapse rate, and NRM incidence in the MAC and RIC groups were 25 vs. 30%, 20 vs. 25%, 33 vs. 32%, and 47 vs. 43%, respectively. In multivariate analysis, the use of bone marrow as a graft source and *ex vivo* T-cell depletion were associated with higher NRM incidence and inferior survival. Furthermore, the intensity of the conditioning regimen was not associated with outcome. The EBMT recently reported the outcome of 324 MCL patients treated with ALLO-SCT between 2000 and 2008. The results are interesting because after a median follow-up of 70 months of a heavily pretreated population (46% of patients received previous AUTO-SCT, 60% of patients received more than 3 CT lines), one-third of patients were progression free. Again, survival was better with chemosensitive disease (54). The toxicity of ALLO-SCT remains important and limits its applicability. Several factors can increase the risk of death due to toxicity. The first is the period in which ALLO-SCT is performed because it is clear that the mortality rate has improved in recent years (55). Indeed, the year of inclusion in the more recently published series (from 2015) ranges from 1999 to 2013 (50, 51, 54, 56). In the last 2 series, the years of inclusion were 2013–2016 and 1999–2013 (57, 58). In Table 9 (10), we present factors predicting NRM incidence from these last studies. Not surprisingly, only severe aGVHD was predictive of a high mortality rate [in 2 studies, (51, 58)], while only one study found age <60 years and heavy pretreatment to be predictive of severe toxicity (58).

**Table 8:** Clinical results of ALLO-SCT in R/R MCL.

	***N***	**CTX**	**Disease status**	**2–5 y OS**	**PFS**	**Relapse rate**	**NRM**
Robinson et al. (1)	22	RIC	CTS 73%	13%	/	100%	82%
Maris et al. (59)	33	NMAC	CTS 54%	64%	60%	16%	24%
Armand et al. (60)	15	RIC	/	42%	22%	33%	37%
Tam et al. (61)	35	RIC	CTS 83%	53%	46%	/	9%
Cook et al. (49)	70	MAC	CTS 83%	37%	14%	65%	18%
Hamadani et al. (53)^§^	202	MAC 74 RIC 128	CTS 0%	25% 30%	20% 25%	33% 32%	47% 43%
Le Gouill et al. (54)	70	RIC	CTS				
Fenske et al. (62)	Early AUTO Early ALLO Late AUTO Late ALLO	RIC	CTS 100%	61% 62% 44% 31%	52% 55% 29% 24%	32% 15% 51% 38%	3% 25% 9% 17%
Kruger et al. (63)	39		CTS 92%	73%	67%	15%	24%
Mussetti et al. (56)	29	RIC	CTS 90%	54%	41%	28%	29%
Vaughn et al. (50)	70	NMAC	CTS 64%	55%	46%	26%	28%
Tessoulin et al. (51)	106	RIC	CTS 80%	62%	43%	30%	28%
Robinson et al. (52)	324	RIC	CTS 65%	40%	31%	40%	24%
Dreger et al. (57)	22	RIC 82%					5%
Arcari et al. (58)	55	RIC	CTS 93%	56%	53%	16%	23%

MAC, myeloablative conditioning regimen; RIC, reduced intensity conditioning regimen; NMAC, nonmyeloablative conditioning regimen. CTS, chemosensitive.

^§^In this study, all patients were chemorefractory at the time of ALLO-SCT.

**Table 9:** Factors predictive of higher NRM incidence in MCL.

	***N***	**CTX**	**Risk factors**	**NRM**
Mussetti et al. (56)	29	RIC	NR	29%
Vaughn et al. (50)	70	NMAC	No factors	28%
Tessoulin et al. (51)	106	RIC	G3–4 aGVHD	28%
Robinson et al. (52)	324	RIC	No factors	24%
Dreger et al. (58)	22	RIC 82%	NR	5%
Arcari et al. (57)	59	RIC	G3–4 aGVHD, > 2 CT lines, age > 60 y	23%

Of note, MIPI, histological and/or molecular variables did not predict the clinical outcome. However, these informations were frequently unknown in registry studies.

## Results after CAR-T cell therapy in MCL

CD19 CAR-T cells have emerged as a highly active treatment modality for R/R MCL. The experience with CAR-T cells in MCL is more limited than that in large B-cell lymphomas.

Table 10 shows the results of two studies: the ZUMA-2 study, a phase 2 prospective trial that led to FDA approval (July 2020) of brexucabtagene autoleucel (brexu-cel), and a real-life retrospective study.

**Table 10:** Clinical results after CAR-T-cell therapy in MCL.

**Authors**	***N* PZ**	**Median age**	**Phase**	**CAR-*T*-cell therapy**	**N CT** **lines**	**TP53** **mutated**	**ORR**	**PFS**	**OS**
Wang et al. (64)	68	65	P 2	Brexu-cel	>3	6/36	85% (CR 59%)	61%	83%
Iacoboni et al. (28)	39	67	R	Brexu-cel	2	12%	91% (CR 79%)	77%	83%

NR, not reached.

In the ZUMA-2 (16) trial, in terms of baseline characteristics, 31% of patients had blastoid or pleomorphic histology, 81% of patients received ≥3 prior lines of treatment, 6 of 36 patients had TP53 mutation (of those with available data), and all patients had prior BTKi treatment (acalabrutinib and/or ibrutinib). Among the 68 treated patients, the 1-y PFS was 61%, with a median PFS not reached at the time of study publication. Subgroup analysis demonstrated a similar ORR and 6-month PFS among high-risk subgroups, including patients with TP53 mutation, patients with blastoid morphology, and patients with high-risk MIPI, compared with patients without these high-risk features (64). Recently, a 3-year follow-up analysis of this study was published. The ORR and CR rate were 91 and 68%, respectively. The duration of response (DOR) for responding patients was 28.2 months, and the median PFS was 25.8 months. Although not significant, a trend toward a lower ORR was observed in the high-risk subgroups (p53 mutation, POD24, and blastoid histology subgroups), even if these data are still unstable due to low number of patients analyzed.

Pretreatment with a BTK inhibitor did not impact the ORR, while exposure to bendamustine seemed to have a negative impact on the DOR (65).

Iacoboni et al. recently published the first real-world study from Europe of brexu-cel in high-risk MCL (high MIPI score, poor Eastern Cooperative Oncology Group performance status and receipt previous allogeneic HCT). The ORR was 91% (CR rate 79%), and the 1-y PFS and OS were 50.8 and 61.4%, respectively. However, in this cohort of patients, the mortality rate related to CAR-T-cell therapy was 15% (28).

The safety profile is quite different for different cellular products (Table 11). The use of liso-cel appears to be associated with a lower incidence of CRS and neurotoxicity than the use of brexu-cel. Grade 5 toxicities occurred in two patients (3%).

**Table 11:** Main toxicities after CAR-T-cell therapy in MCL.

	**CRS**				**ICANS**				**Cytopenia**	
	**Brexu-cel**		**Liso-cel**		**Brexu-cel**		**Liso-cel**		**Brexu-cel**	**Liso-cel**
	**Any**	**Sever**	** Any**	**Severe**	**Any**	**Severe**	**Any**	**Severe**		
Wang et al. (65)	91%	15%			63%	31%			26%	
Iacoboni et al. (28)	91%	16%			91%	3%			50% at 1 month	

Severe toxicities were defined as those ≥ grade 3.

In light of the results described above, the ideal R/R MCL patients for CAR-T-cell treatment are those with progressive disease following BTKi therapy, but these patients be well enough physiologically to tolerate expected complications, including CRS, and those patients with significant frailty or with severe end organ damage, such as severe systolic heart failure, should generally not be candidates for this type of therapy.

## Possible scenarios for integrating ALLO-SCT and CAR-T-Cell therapy

Although it is now widely acknowledged that CAR-T-cell therapy is useful in DLBCL and MCL and thus that ALLO-SCT does not have a place in the treatment scheme, we do not think that these 2 immunological approaches to cure advanced lymphoma are mutually exclusive (66). In the recent European Bone Marrow Transplantation Society (EBMT) guidelines, the role of ALLO-SCT was modified: it is now considered only an option, while CAR-T-cell therapy is the standard of care (67).

Three clinical scenarios can be proposed: first, patients can be treated with ALLO-SCT before CAR-T-cell therapy; second, CAR-T-cell therapy can be applied first, and ALLO-SCT can be applied if there is progression/relapse; and third, CAR-T-cell therapy can be used as induction therapy in a tandem CAR-T-cell therapy/ALLO-SCT sequence, as frequently done for acute lymphoblastic leukemia.

The first scenario has already been proved lack utility, and in different countries, CAR-T cells are already approved by regulatory agencies as the first-line immunotherapy in R/R LBCL and MCL with specific indications. Dreger et al. compared the results obtained in an intention-to-treat analysis of patients with R/R LBCL at their centre. In the first period (2004–2020), ALLO-SCT was considered the preferred option (*n* = 60), while in the second period (2018–2020), CAR-T-cell therapy was considered the standard of care (*n* = 41). The researchers did not observe differences in terms of 1-y OS (68 vs. 54%), 1-PFS (39 vs. 33%) or relapse incidence (59 vs. 44%), but there was a significant difference in NRM incidence in favor of CAR-T-cell therapy (3 vs. 21%) (68). More recently, in a registry study, the Center for International Blood and Marrow Transplant Research (CIBMTR) analyzed results obtained from patients with DLBCL who relapsed after autologous transplantation and were treated with ALLO-SCT or CAR-T-cell therapy (axi-cel). In the CAR-T cell therapy cohort, at 1 year, the relapse rate was 39.5%, the NRM incidence was 4.8%, the OS was 73.4%, and the PFS was 55.7%. In the ALLO-SCT group, the results were similar, except for NRM incidence (26.2, 20, 65.6, and 53.8%, respectively). The clinical characteristics were different mainly in terms of disease status at the time of cellular therapy, as only 26% of patients in the CAR-T-cell therapy group had a disease status of CR or PR, compared to 80% in the ALLO-SCT group. Furthermore, in that study, the CIBMTR score applied to 2 cohorts clearly separated three groups of patients with different survival (20). However, in patients in CR/PR after bridging therapy, considering that the relapse rate is low after allo-SCT, in presence of p53 mutation, this kind of therapy should be considered.

For R/R MCL, similar to LBCL, the scenario has also already been shown to be ineffective. In the recent American Society of Transplantation and Cellular Therapy, CIBMTR, and EBMT clinical practice recommendations for cellular therapies in MCL, CAR-T-cell therapy is recommended as the standard of care for patients with R/R MCL (69).

In the second scenario, CAR-T-cell therapy is used as induction therapy to perform ALLO-SCT. However, it is getting easier to predict the outcome of a single patient after CAR-T-cell therapy based on several predictive factors before and after infusion. In our opinion, one of the most interesting predictive factors is lack of complete remission at disease response evaluation 1–3 months after infusion, as reported by Nastoupil et al. (25). This factor can be combined with other factors, such as expansion of CAR-T cells early after infusion. For these high-risk patients, ALLO-SCT could be used after reinduction therapy to obtain CR or to reduce the lymphoma burden as much as possible. However, specific studies should be conducted in this field, using strong predictive factors of post-CAR-T cells outcome.

For both lymphoma subtypes, the 3rd scenario, in which CAR-T cell therapy is the first-line choice in refractory patients and ALLO-SCT is reserved for relapsed patients, provided that a clinical response is obtained, is probably more realistic, as reported in a recent survey by ASTCT (70). In this survey, the majority of physician considers allo-SCT in patients failing CAR-T and responding to salvage treatment. Shadman et al. first reported the outcomes of 13 patients who relapsed after CAR-T-cell therapy and underwent ALLO-SCT. Although the NRM incidence was relatively high (33% at 100 days), in part due to the use of a myeloablative conditioning regimen (39% of patients), the 1-year OS was encouraging (59%) (71). Chow et al. analyzed the outcomes of 61 patients who relapsed and progressed early (in the first 30 days, *n* = 26) or late (*n* = 35) after CAR-T-cell infusion. One-quarter of the patients did not receive any treatment at the time of progression for several reasons. Overall, only 6 patients underwent ALLO-SCT. The median OS of the entire population was only 5.3 months (72). More recently, Zurko et al. reported on 88 patients treated with ALLO-SCT after failing CAR-T-cell therapy. The median time between CAR-T-cell therapy and ALLO-SCT was 255 days (range 63–753). The majority of patients were chemosensitive at the time of ALLO-SCT (76%). After a median of 1 treatment line, reduced intensity conditioning regimens were used in 77% of patients, and there was similar use of various donor types throughout the cohort (MSD 26%, haploidentical 30%, matched unrelated donor 39%). At 100 days after ALLO-SCT, the cumulative incidence rates of grade II-IV and III-IV aGVHD were 34 and 10%, respectively. At 1 year, the cumulative incidence of moderate/severe cGVHD was 7.8 and 3.8%, respectively. The 1-year NRM was 22%, and the 1-year OS and PFS were 59 and 55%, respectively. In the multivariate analysis, the number of lines of therapy between CAR-T-cell therapy and ALLO-SCT and disease status at the time of ALLO-SCT were the most important prognostic factors for survival (73).

Di Blasi et al. (74) recently reported on 238 patients relapsed/refractory after CAR-T (both axi-cel and tisa-cel) in France. Relapse/progression was classified as very early (before d +30 days after CAR-T), early (between d +31 and d+90), and late (> d +90). Information on therapies received was available in 64% of patients, and mostly received lenalidomide (38%), target therapy (21%) and immune-chemotherapy (20%). The overall response rate was 14% (CR rate 65) and the median survival range from 3.7 and 8.5 months. In the multivariate analysis, predictive factors for PFS were LDH and ferritin levels at infusion, and for OS LDH, CRP, and very early relapse. To note, no association with outcome was observed for treatment type. This study confirms that R/R NHL after CARE-T is an unmet clinical need.

Furthermore, the toxicity observed after these two kinds of immunotherapy is deeply different, because allo-SCT is still complicated by a significant NRM due to infections and GVH, while the safety profile of CAR-T is acceptable. Of course, this should be take in account planning to treat the patients with adoptive immunotherapy.

In conclusion, even if ALLO-SCT for patients who relapse/progress after CAR-T seems reasonable, this population is very difficult to treat. Furthermore, ALLO-SCT can be complicated by the aggressiveness of disease, poor patient performance status and/or cytopenias, which can preclude the administration of induction therapy.

## Author contributions

LC and RB wrote the manuscript. ST, GS, AI, AS, VT, LN, AM, and CP revised the manuscript. All authors contributed to the article and approved the submitted version.

## Conflict of interest

The authors declare that the research was conducted in the absence of any commercial or financial relationships that could be construed as a potential conflict of interest.

## Publisher's note

All claims expressed in this article are solely those of the authors and do not necessarily represent those of their affiliated organizations, or those of the publisher, the editors and the reviewers. Any product that may be evaluated in this article, or claim that may be made by its manufacturer, is not guaranteed or endorsed by the publisher.
